# Preoperative DSA-guided embolization combined with surgical resection for the treatment of intratumoral hemorrhage in plexiform neurofibroma: a case report

**DOI:** 10.3389/fonc.2026.1826614

**Published:** 2026-09-23

**Authors:** Yuzhe Shi, Ziqiong Wang, Qiwen Chen, Yuxin Chen, Chuyue Zhong, Zeru Xu

**Affiliations:** 1 Department of Breast Surgery, The First Affiliated Hospital of Wenzhou Medical University, Wenzhou, Zhejiang, China; 2 The Second Affiliated Hospital and Yuying Children’s Hospital of Wenzhou Medical University, Wenzhou, Zhejiang, China; 3 Department of Gastroenterology, The First Affiliated Hospital of Wenzhou Medical University, Wenzhou, Zhejiang, China; 4 Department of Wound Repair, The First Affiliated Hospital of Wenzhou Medical University, Wenzhou, Zhejiang, China

**Keywords:** digital subtraction angiography, intratumoral hemorrhage, multidisciplinary management, neurofibromatosis type 1, plexiform neurofibroma

## Abstract

**Background:**

Neurofibromatosis type 1 (NF1) is a neurocutaneous genetic disorder. Plexiform neurofibromas (PNFs), occurring in a subset of patients, carry a risk of significant hemorrhage, which can be life-threatening.

**Case presentation:**

A 61-year-old woman with NF1 presented with a rapidly enlarging, painful left buttock mass after trauma. Imaging confirmed a PNF with intratumoral hemorrhage. She underwent preoperative digital subtraction angiography (DSA) with embolization of tumor-feeding arteries, followed by successful complete surgical resection. At 6-month follow-up, the patient remained free of recurrent hemorrhage, with satisfactory wound healing and significant improvement in pain and mobility.

**Discussion:**

Giant plexiform neurofibromas complicated by acute hemorrhage represent a rare but clinically challenging manifestation of NF1. This case demonstrates that a staged strategy combining preoperative DSA-guided embolization and surgical resection within a multidisciplinary framework can effectively control bleeding and optimize perioperative safety. Comprehensive risk assessment, including vigilance for thromboembolic complications, is essential in high-risk surgical patients. Given the lifelong risk of malignant transformation, long-term surveillance and consideration of targeted therapy remain critical, although real-world accessibility may limit implementation.

**Conclusion:**

Preoperative embolization followed by resection represents a safe and effective multidisciplinary strategy for controlling acute, life-threatening hemorrhage in NF1-associated PNF. Similarly, perioperative surveillance and long-term follow-up remain essential for achieving optimal clinical outcomes.

## Introduction

Neurofibromatosis-related disorders are classified into three distinct types based on clinical and genetic criteria: neurofibromatosis type 1 (NF1), type 2 (NF2), and schwannomatosis. NF1, also known as von Recklinghausen disease, is a relatively common autosomal dominant disorder with an estimated incidence of 1 in 2,500–3,000 live births. It is caused by pathogenic variants in the NF1 gene on chromosome 17q11.2 (1, 2). This multisystem condition is characterized by café-au-lait pigmentation, freckling in intertriginous regions, multiple cutaneous neurofibromas, and variable degrees of learning or behavioral impairment. Plexiform neurofibromas (PNFs) occur in approximately 25–50% of patients with NF1 and may arise along peripheral nerves or nerves plexuses. Notably, PNFs carry an 8–13% lifetime risk of malignant transformation into malignant peripheral nerve sheath tumors (MPNSTs) (1, 3). PNFs are also prone to significant hemorrhage due to fragile intratumoral vasculature, which can occur spontaneously or after minor trauma, with reported cases involving the scalp, face, and buttock, which can pose life-threatening risks (4–6).

We report a case of NF1 with severe intratumoral hemorrhage within a giant PNF involving the buttock, successfully managed with preoperative embolization and surgical resection.

## Case presentation

### Preoperative evaluation

A 61-year-old woman with known NF1 presented with rapid enlargement and severe pain in a left buttock mass following a fall one month prior. Her vital signs were stable. Laboratory tests showed an elevated D-dimer level of 0.94 mg/L. Past surgical history included resection of neurofibromas over 30 years ago and a hysterectomy six years ago. Physical examination revealed multiple café-au-lait macules and cutaneous neurofibromas scattered across the torso and limbs. Two distinct PNFs were noted: one in the sacrococcygeal region (~18 × 13 cm, non-tender, “bag of worms” consistency) and another in the left buttock (~30 × 22 cm, tender, with overlying erythema and increased skin tension) (**Figure 1A**).

**Figure 1:**
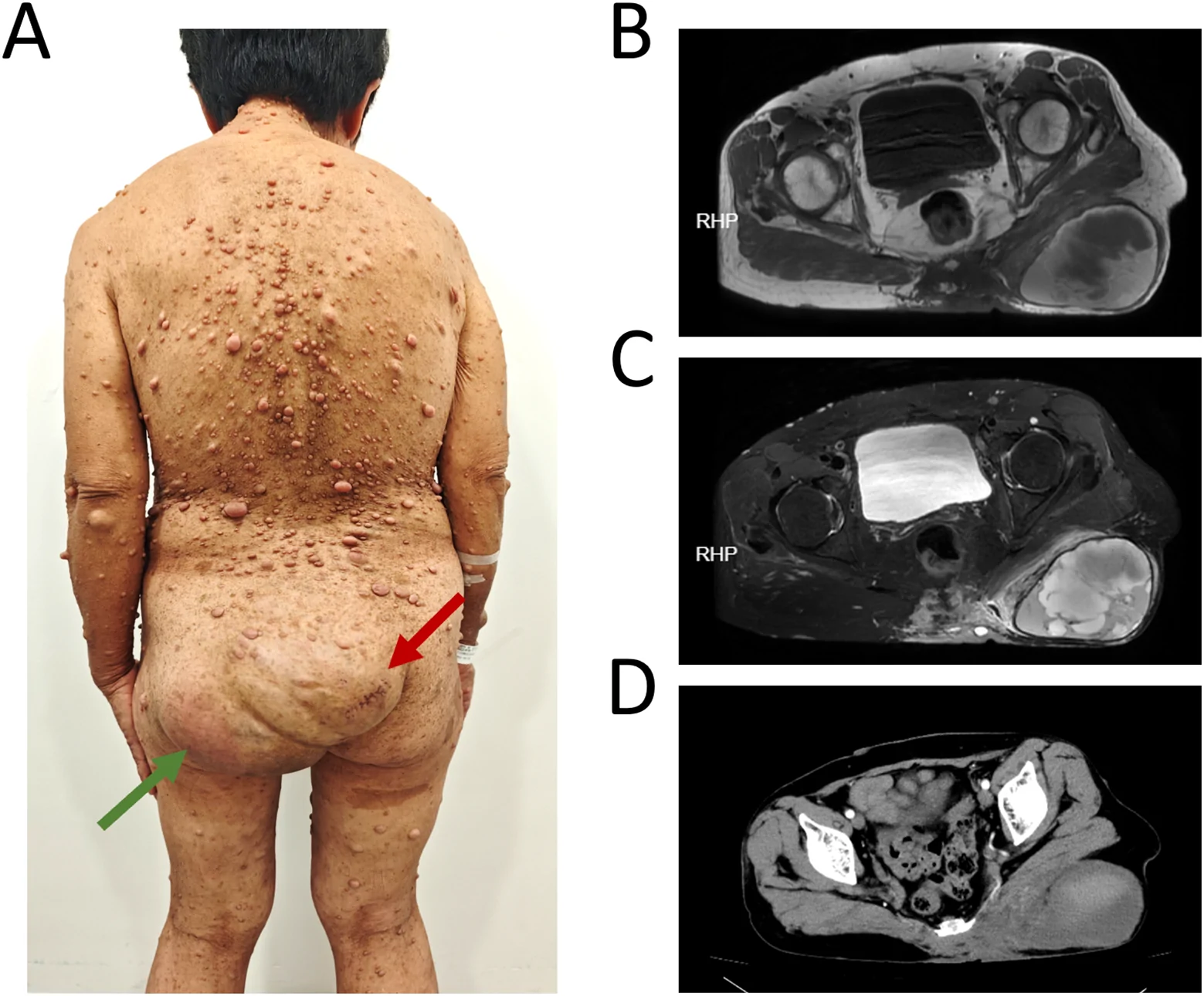
Clinical and radiological findings of the lesion. **(A)** Clinical photograph of the patient from posterior view. Multiple café-au-lait macules and cutaneous neurofibromas are visible and scattered across the torso and limbs. Two distinct PNFs are observed: one in the sacrococcygeal region (~18×13 cm, red arrow) and another in the left buttock (~30×22 cm, green arrow). **(B)** Axial T1-weighted MRI shows a heterogeneous subcutaneous mass with mixed signal intensity. **(C)** Axial T2-weighted MRI demonstrates a predominantly hyperintense lesion with surrounding edema. **(D)** Contrast-enhanced CT reveals a cystic mass containing patchy high-density areas and no obvious enhancement.

MRI of the left buttock demonstrated a heterogeneous subcutaneous mass with mixed T1 and high T2 signal intensity and surrounding edema (**Figures 1B, C**). Contrast-enhanced CT revealed a cystic, mass-like lesion containing patchy high-density areas without significant enhancement. The imaging findings confirmed the presence of an intratumoral hemorrhage and an expanding hematoma within the pre-existing PNF (**Figure 1D**).

### Preoperative embolization

Given the large size and rich vascularity of the PNF in the left buttock, blind surgical resection could have led to massive intraoperative hemorrhage, potentially endangering the patient’s life. Therefore, preoperative identification and embolization of the tumor feeding arteries under digital subtraction angiography (DSA) was considered the optimal approach. Under local anesthesia, the right femoral artery was punctured using the Seldinger technique. DSA revealed a mildly hypervascular lesion in the left buttock, with enlargement and increased distal branching of the left superior gluteal artery and the left third and fourth lumbar arteries (**Figures 2A, C**). Embolization of these arteries was performed using gelatin sponge particles and gelatin sponge strips. Follow-up DSA confirmed satisfactory embolization (**Figures 2B, D**). The patient experienced no postoperative complications.

**Figure 2:**
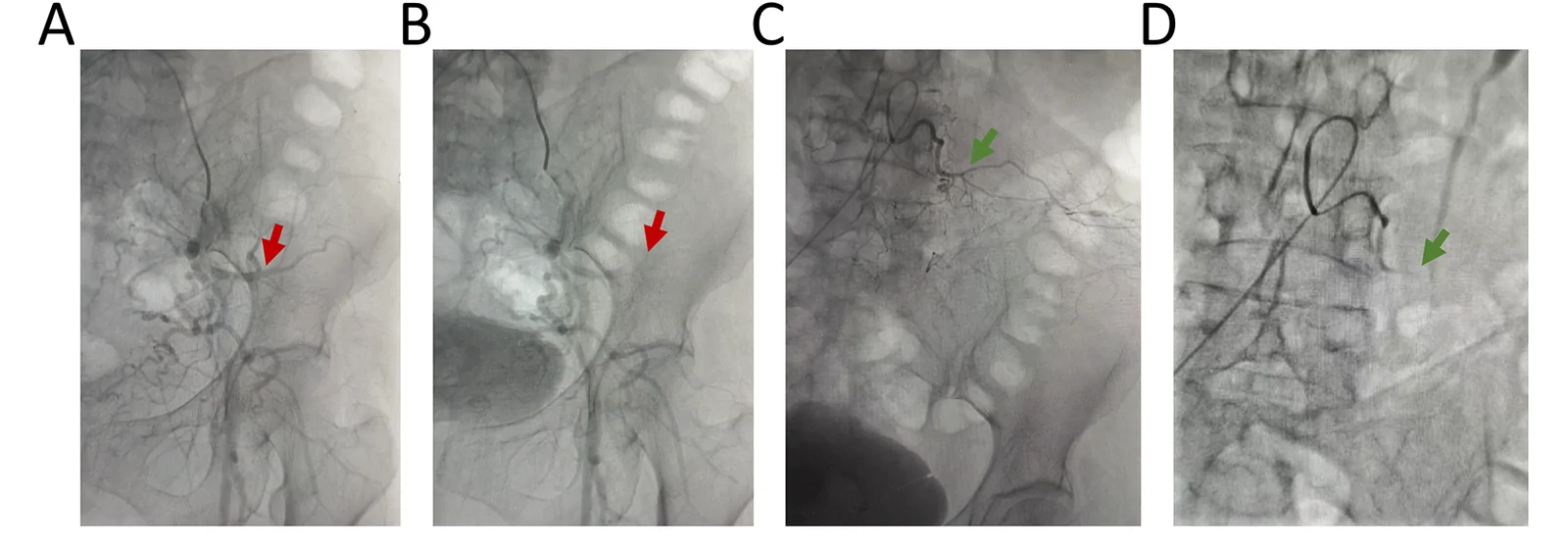
Preoperative digital subtraction angiography (DSA) and transarterial embolization. **(A)** Pre-embolization DSA of the left superior gluteal artery (red arrow). **(B)** Post-embolization DSA of the left superior gluteal artery (red arrow) showing successful occlusion of the vessel. **(C)** Pre-embolization DSA of the left third and fourth lumbar arteries (green arrows). **(D)** Post-embolization DSA of the left third and fourth lumbar arteries (green arrows) confirming complete devascularization.

### Surgical resection and postoperative course

On the day following DSA, the patient was placed in the prone position under general anesthesia with endotracheal intubation. A skin incision was made down to the fascia, and the superficial hematoma was evacuated. The tumor, adherent to the gluteus maximus, was bluntly dissected and excised en bloc along with the overlying skin and soft tissue, and the specimen was subsequently sent for histopathological examination. Hemostasis was achieved, the surgical site was irrigated, a drain was placed, and the wound was closed in layers (**Figures 3A–E**). The procedure was uneventful, and the patient’s vital signs remained stable throughout.

**Figure 3:**
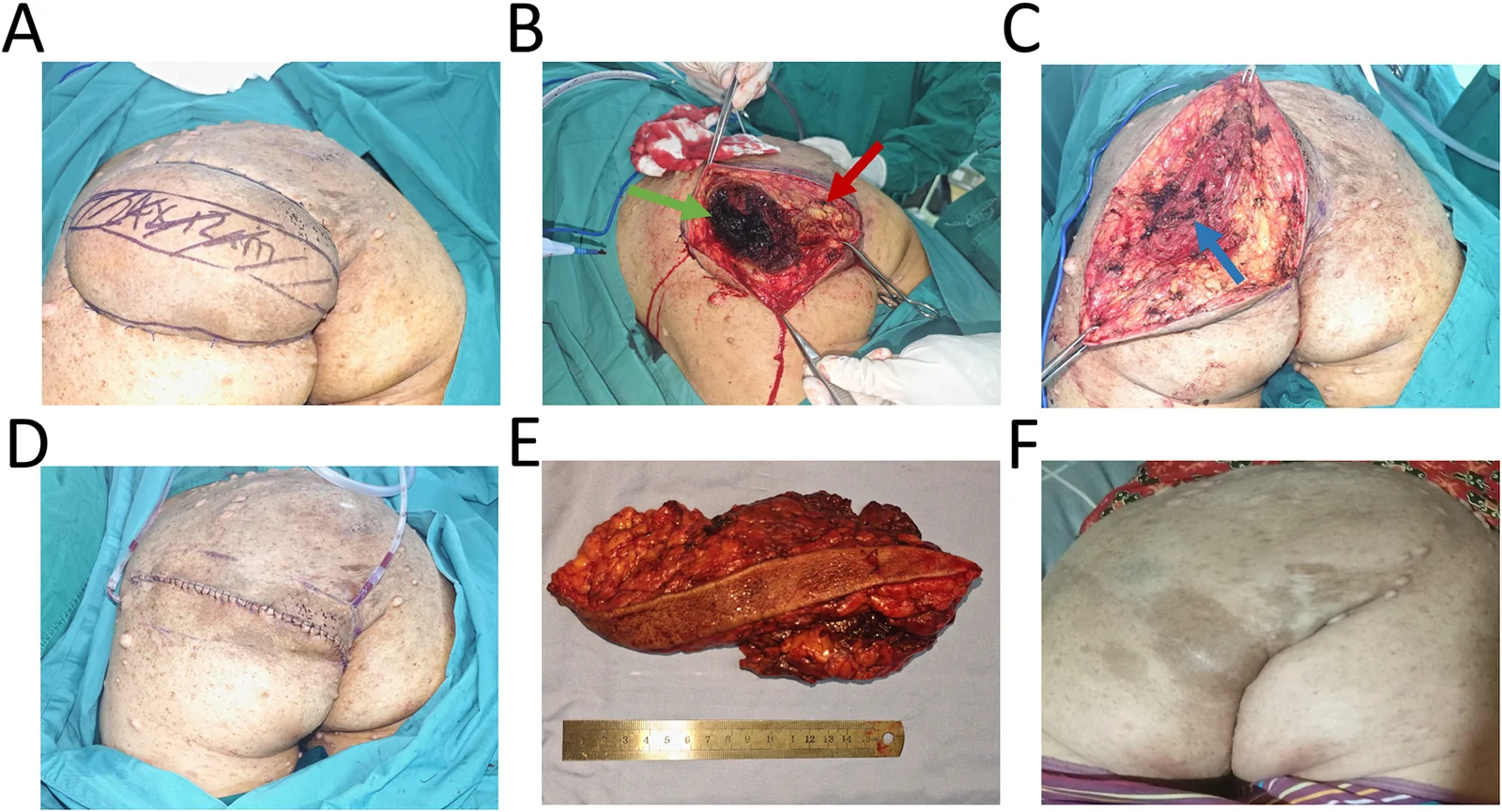
Key steps of the surgical resection. **(A)** Preoperative appearance of the mass. **(B)** Intraoperative view following skin incision, showing the superficial hematoma (green arrow) and the tumor (red arrow). **(C)** Surgical field after complete en bloc resection of the tumor, with the gluteus maximus indicated by the blue arrow. **(D)** Closure of the surgical wound in layers with placement of a drain. **(E)** Photograph of the resected specimen, including the tumor with overlying skin and soft tissue, which was sent for histopathological examination. **(F)** Clinical photograph obtained 6 months after surgery, demonstrating satisfactory wound healing and favorable postoperative recovery.

On postoperative day 1, the dressing was changed, and the wound demonstrated good healing without evidence of hematoma or infection. The drain was removed on postoperative day 5, and the patient was discharged on day 7. The timeline of events during the patient’s hospitalization is shown in **Figure 4**. Histopathological immunohistochemistry showed Ki-67 (1–2%+), p53 (wild-type), S-100 (+), and Sox-10 (+), supporting the diagnosis of neurofibroma. At the 3-week follow-up, the patient reported no discomfort, and the wound had healed well. At the 6-month follow-up, the surgical wound remained well healed without evidence of recurrent hemorrhage, infection, or neurological deficit (**Figure 3F**). The patient reported significant improvement in pain and mobility and was satisfied with the treatment outcome.

**Figure 4:**
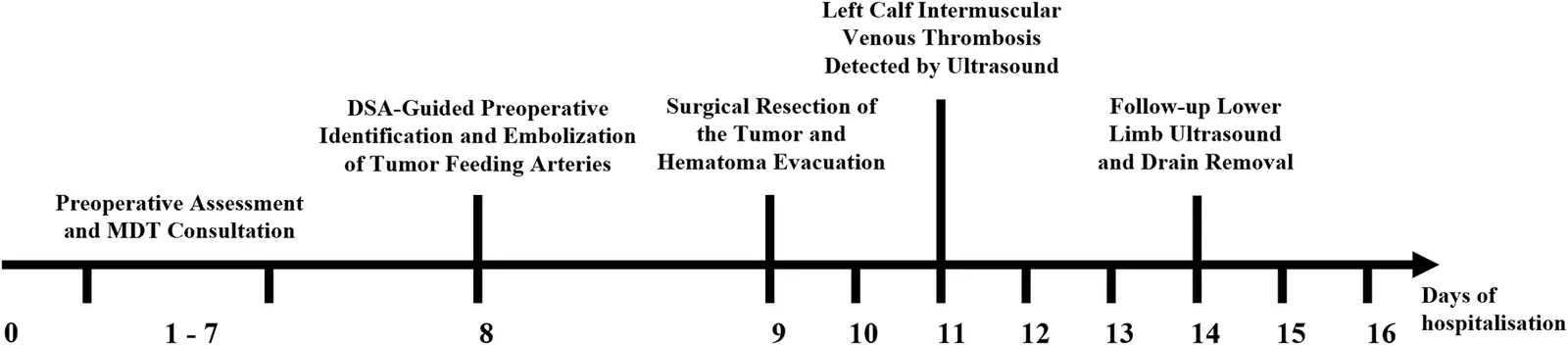
Timeline of events during hospitalization.

### Patient perspective

The patient reported that the rapidly enlarging mass and associated pain caused substantial anxiety and impairment in daily activities. Following embolization and surgical treatment, she experienced marked symptom relief and was satisfied with the recovery process. She expressed appreciation for the coordinated multidisciplinary care provided during hospitalization.

## Discussion

This case highlights the complex management of neurofibromatosis type 1 (NF1) complicated by a giant plexiform neurofibroma (PNF) with acute hemorrhage. Beyond urgent bleeding control in a highly vascular tumor, care required perioperative risk assessment, multidisciplinary coordination, thromboembolic surveillance, and long-term monitoring for malignant transformation. A staged interventional–surgical approach within an Multidisciplinary Team (MDT) framework optimized safety while underscoring the challenges of targeted therapy and real-world treatment accessibility.

Although preoperative embolization followed by surgical resection has been described in previous reports of hemorrhagic PNFs (5), the present case provides several additional clinical insights. First, the hemorrhage occurred within a giant gluteal PNF following trauma, an uncommon presentation with substantial surgical complexity. Second, management extended beyond hemorrhage control and involved coordinated multidisciplinary decision-making across several specialties. Third, this case highlights the importance of perioperative VTE surveillance and long-term risk management, including assessment of malignant transformation risk and consideration of targeted therapy. These aspects may provide practical guidance for clinicians managing similarly complex NF1 patients.

### Vascular complications in NF1 and management of acute hemorrhage

NF1 arises from mutations in the NF1 gene, leading to a loss of function of neurofibromin, a protein pivotal in regulating cell proliferation and maintaining vascular integrity (7). Although vascular abnormalities are observed in only a minority of patients (<3%) (8), they can manifest as arterial stenosis, aneurysms, or arteriovenous malformations, and are notorious for their potential for spontaneous and catastrophic rupture (9). Fatal hemorrhages due to spontaneous rupture of visceral vessels, such as primary subarachnoid hemorrhage, spontaneous hemothorax, or renal artery rupture, have been extensively documented in the literature (10–13). In contrast, massive hemorrhage originating directly from a giant PNF, while potentially carrying a lower immediate risk of exsanguinating shock, can lead to severe complications through the compressive effects of the resulting hematoma on adjacent structures (4, 14), as demonstrated in the present case. Unlike the spontaneous rupture of visceral vessels, hemorrhage from a giant PNF may permit a more controlled, staged therapeutic approach. We successfully employed preoperative DSA for the precise identification and subsequent embolization of the tumor-feeding arteries. This strategy established a safer surgical window, minimized intraoperative blood loss, and facilitated the complete en bloc resection of the tumor along with the overlying involved skin and soft tissue on the following day.

### The role of MDT management in complex NF1 patients

The multidisciplinary diagnostic and treatment guidelines for neurofibromatosis type 1 (NF1) emphasize that although NF1 is primarily characterized by neurofibromas, it frequently involves multiple organ systems and presents substantial challenges in diagnosis, treatment, follow-up, and long-term management. Therefore, the establishment of an MDT approach is essential. Particularly in complex NF1 cases, MDT management enables integration of expertise from radiology, interventional radiology, neurology, neurosurgery, and oncology to formulate individualized treatment strategies. Several published case reports have demonstrated that MDT-based management can optimize therapeutic processes and improve patient outcomes (15–17).

The present case further illustrates the value of MDT collaboration. Preoperatively, radiologists conducted comprehensive evaluations of MRI and CT imaging to delineate the extent of hemorrhage and characterize tumor vascularity, while neurologists performed systematic neurological assessments to inform surgical risk evaluation. During treatment, the interventional team precisely embolized the tumor-feeding arteries, creating safer operative conditions for subsequent resection. The wound repair team then performed tumor excision along with resection of involved tissues and managed the surgical defect. Postoperatively, vascular surgeons participated in thrombotic risk assessment and management, and oncologists developed a long-term follow-up and potential targeted therapy plan. Close interdisciplinary communication and coordinated decision-making were critical in ensuring perioperative safety and favorable long-term prognosis.

### Risk identification and management of perioperative venous thromboembolism

It is noteworthy that on postoperative day 2, lower limb vascular ultrasonography detected an intermuscular venous thrombosis in the left calf. Given the elevated risk of bleeding in the early postoperative period, anticoagulation therapy was deferred after comprehensive evaluation. Instead, strict lower limb immobilization and dynamic ultrasonographic surveillance were implemented to reduce the risk of thrombus migration. Repeat lower limb vascular ultrasonography on postoperative day 5 demonstrated persistence of the thrombus, although its diameter had decreased. Upon discharge, the patient was advised to continue restricted lower limb activity and undergo regular vascular ultrasound follow-up.

In retrospect, advanced age, prolonged operative duration, and reduced postoperative mobility may have contributed to the increased risk of deep vein thrombosis (DVT) in this patient. Routine perioperative lower limb vascular ultrasonography may facilitate early detection and enable timely individualized intervention. In addition, for surgical patients at high risk of DVT, the intraoperative or postoperative application of intermittent pneumatic compression (IPC) devices represents an important mechanical prophylactic strategy. A meta-analysis including 16 randomized controlled trials reported that IPC reduced the risk of DVT (OR = 0.81, 95% CI: 0.59–1.11). Although statistical significance was limited, these findings suggest a potential benefit of IPC in high-risk populations.

### Risk of malignant transformation and the dilemma of targeted therapy

Beyond acute complications, PNFs carry a lifetime risk of malignant transformation into MPNST, estimated between 8% and 13% (1, 18). Clinical indicators suggesting malignant change include rapid tumor growth, new onset of pain, or an irregular contour (19). Selumetinib is a potent, selective, allosteric inhibitor of MEK1/2. Its binding to MEK1/2 inhibits the phosphorylation of ERK1/2, thereby suppressing downstream pathways involved in cell proliferation, survival, differentiation, and apoptosis (20, 21). Multiple clinical trials have demonstrated that selumetinib effectively reduces tumor volume in both pediatric and adult NF1 patients with PNF, alleviates symptoms, improves quality of life, and exhibits a favorable safety profile (22–24). In this case, a comprehensive multidisciplinary assessment did not reveal signs indicative of PNF deterioration. Because an additional sacrococcygeal plexiform neurofibroma remained after surgery, the patient was advised to consider selumetinib therapy for residual disease control and long-term tumor management. Regrettably, the patient declined the pharmacological treatment due to financial constraints, highlighting a common barrier to optimal targeted therapy in real-world practice.

### Strengths and limitations

A major strength of this case was the staged combination of DSA-guided embolization and surgical resection within a multidisciplinary framework, which enabled effective hemorrhage control while minimizing perioperative risk. This report has several limitations. First, it describes a single patient and therefore may not be generalizable to all NF1-associated hemorrhagic PNFs. Second, postoperative MRI was not available to provide radiological confirmation of complete lesion removal. Third, long-term follow-up data remain limited. Larger studies are needed to further evaluate the effectiveness and safety of multidisciplinary management strategies in these patients.

## Conclusion

For NF1 patients presenting with life-threatening intratumoral hemorrhage, a multidisciplinary approach is crucial. Preoperative embolization of feeding arteries followed by surgical resection represents a safe and effective treatment strategy to control bleeding and achieve complete tumor removal. In addition, careful perioperative surveillance and long-term follow-up are essential to optimize clinical outcomes and support ongoing disease management.

## Data Availability

The original contributions presented in the study are included in the article/supplementary material. Further inquiries can be directed to the corresponding author.
